# Curiosities of Life

**Published:** 1871-05

**Authors:** 


### CURIOSITIES OF LIFE.

Lay your finger on your pulse, and know that at every stroke some immortal passes to his Maker; some fellow-being crosses the river of death; and if we think of it, we may well wonder that it should be so long before our turn comes.

Half of all who live die before seventeen.

Only one person in ten thousand lives to be a hundred years old, and but one in a hundred reaches sixty.

The married live longer than the single.

There is one soldier for every eight persons, and out of every thousand born only ninety-five weddings take place.

If you take a thousand persons who have reached seventy years, there are of

| | |
|---|---|
| Clergymen, orators, and public speakers . . . | 43 |
| Farmers . . . . . . . . . . | 40 |
| Workmen . . . . . . . . . . | 33 |
| Soldiers . . . . . . . . . . | 32 |
| Lawyers . . . . . . . . . . | 29 |
| Professors . . . . . . . . . . | 27 |
| Doctors . . . . . . . . . . | 24 |

These statements are very instructive. Farmers and workmen do not arrive at a good old age as often as the clergymen and others who perform no manual labor; but this is owing to neglect of the laws of health, inattention to proper habits of life in eating, drinking, sleeping, dress, and the proper care of themselves after the work of the day is done. These farmers or workmen eat a hearty supper of a summer's day and sit around the doors in their shirt-sleeves; and in their tired condition and weakened circulation are easily chilled, laying the foundation for diarrhœa, bilious colic, lung-fever, or consumption.

